# Supplementary material for: Evidence of an active volcanic heat source beneath the Pine Island Glacier
Source: Nat Commun. 2018 Jun 22;9:2431. doi: 10.1038/s41467-018-04421-3 (PMC6014989; doi:10.1038/s41467-018-04421-3)
Supplement: Supplementary file 1 — Supplementary Information [file 41467_2018_4421_MOESM1_ESM.pdf]

Supplemental Material for: Evidence of an active volcanic heat source beneath the Pine Island Glacier

Loose et al.,

Supplementary Note 1: Geochemistry of Circumpolar Deep Water

In addition to heat, CDW is easily identified by its $^3He/^4He$ isotope ratio, which accumulates during its transit over mid-ocean ridges in the Atlantic, and Pacific [1], as well as the Southern Ocean [2,3], including along the Pacific-Antarctic Ridge [4]. The light isotope of helium (^3He) is most abundant in the Earth's mantle. This 'primoridial' helium dates to Earth's formation, and volcanism has produced an irreversible degassing of ^3He from the mantle to space [5,6]. The $^3He/^4He$ isotope ratio in the atmosphere (R_A) is 1.384x10^{-6}, and models suggest that the $^3He/^4He$ in the mantle 4.4 Gy ago was ~ 230 R_A, close to the value in gas-rich meteorites [7]. Presently, the largest excess in ^3He is associated with island arc magmatism [~72 R_A , *Class and Goldstein*, 2005], and in oceanic ridge basalts (~8 R_A). Values of $^3He/^4He$ less than 2R_A, e.g. in seawater are commonly expressed using delta notation; $\delta^3\text{He} = (R_{obs}/R_A - 1)\times 100$, where R_{obs} is the observed $^3He/^4He$.

The δ^3He signal that accumulates in CDW stands out against all other water types that are found on Antarctic shelves. This has made it a valuable tracer for observing heat fluxes [8], water mass modifications [9,10], and ocean-derived melting of glaciers [2]. Directly above hydothermal vents, the concentration of δ^3He can be as high as 35%, but the far-field δ^3He values are closer to 10.5% in the Southern Ocean [3].

CDW is modified through ventilation to varying degrees; in the Ross Sea the CDW is heavily ventilated and is often a few tenths of a degree below zero by the time it reaches the Ross Ice Shelf [11]. CDW in the Amundsen Sea is more lightly modified and can penetrate along troughs to reach ice shelves [12] at temperatures that range from θ = 0.5 to 1.2 °C and S > 34.6 [13].

Supplementary Note 2: Review of mantle heat flux estimates

How does the heat flux estimate from beneath the Pine Island Glacier compare to the energy liberated from present day volcanoes? A survey of 51 dormant or quiescent volcanoes indicates they release less than 500 MW of heat energy; at an average of 97 MW [14,15]. Spectral radiance emitted in the IR frequency was used to estimate heat flux from 31 of the 51 estimates [15], and these average to 53 MW. The remainder were derived from thermal budgets of volcanic crater lakes including, Poas volcano in Costa Rica [16,17], and Solfatara volcano in Italy, which demonstrated that diffuse water vapor transport, fumaroles, and geysers can contribute an additional 50 and 1000 MW, beyond heat transfer to the crater lake [18]. After accounting for these other terms, the average volcanic heat energy flux from thermal budget analysis is 180 MW.

Heat flux from active volcanoes is at least ten times greater. The total flux emanating from Grimsvotn volcano on Iceland is 4250 MW [19], and active magma convection prior to the 1977 eruption of Nyiragongo volcano in the Democratic Republic of Congo temporarily released 16,000 MW of heat energy [20].

Whereas the dormant volcanoes release 100's of MW of heat, submarine vent fields along active mid-ocean ridges can release 1000's of MW or more. The Southern Symmetrical Segment and the Endeavor Segment of the Juan de Fuca ridge produce heat fluxes of 1700 and 580 MW, respectively [21]. The Lucky Strike Field along the East Pacific Rise produces 3800 MW of heat energy through smokers and hydrothermal vents [22]. All of the heat energy estimates summarized here can be found in Table S3.

Table 1: The end member values and their uncertainties used in the OMP linear mixing model. CDW refers to Circumpolar Deep Water, ASW is short for Air-Satured Water, and GMW is Glacial Meltwater.

	CDW	ASW	GMW
**θ (°C)**	1.09 ± 0.1	-1.86 ± 0.51	-90
**S (ppt)**	34.67 ± 0.41	34.1 ± 0.13	0
**Ne (μmol kg^{-1})**	(8.25 ± 0.2) x 10^{-4}	(8.3 ± 0.825) x 10^{-4}	(8.96 ± 0.8) x 10^{-2}
**δ^3He (%)**	9.15 ± 0.65	0.4 ± 2.1	0

Table 2: The ratio of ^{3}He/heat (10^{16} Joules per mol) from sub-seafloor vents.

**Authors**	**Description**	**^{3}He/heat ratio (10^{17} Joules per mol)**	
		Lower	**Upper**
Jenkins et al., (1978)[1]	Galapagos vents, 4 fields including 'Garden of Eden'	19.2	23.9
Jean Baptiste (2004) [23]	Rainbow vent		11.1
Lupton et al (1989) [24]			25.0
Lupton (1999)[25]	Event plumes (not steady state)		25.0
	East Pacific Rise, 21°N	16.4	23.3
	Galapagos Rift		19.2
	East Pacific Rise, 13°N	6.8	14.1
	Cleft Segment		18.9
	TAG 26°N	7.7	20.0
	Snake Pit 23°N	7.7	17.5
	Lucky Strike 37°N	13.0	19.6
Jenkins (2015)[26]	Geotraces from North Atlantic section		14.3
	**Average**	**17 ± 6**	

Table 3: Inventory of heat energy in Megawatts (MW) from dormant, active and sub-sea volcanoes.

Authors	Notes	Volcano	State	Bulk Flux (MW)	
				Lower	Upper
Wright and Flynn, Geology 2004[15]	Remote sensing of black body radiation	Ambrym	Dormant	1.26	39.95
		Arenal	Dormant	0.37	11.73
		Bagana	Dormant	0.62	19.66
		Belinda	Dormant	0.06	1.90
		Bezymianny	Dormant	0.33	10.46
		Big Ben	Dormant	0.01	0.32
		Cleveland	Dormant	0.07	2.22
		Erebus	Dormant	0.6	19.03
		Erta ‘Ale	Dormant	2.24	71.03
		Etna	Dormant	7.93	251.46
		Ibu	Dormant	0.2	6.34
		Karangetang	Dormant	0.78	24.73
		Kilauea	Dormant	13	412.23
		Krakatau	Dormant	0.27	8.56
		Lascar	Dormant	0.44	13.95
		Lopevi	Dormant	0.16	5.07
		Mayon	Dormant	0.3	9.51
		Merapi	Dormant	2.13	67.54
		Michael	Dormant	0.37	11.73
		Nyamuragira	Dormant	13.3	421.74
		Pacaya	Dormant	0.01	0.32
		Piton de la Fournaise	Dormant	1.71	54.22
		Popocatepetle	Dormant	0.71	22.51
		Rabaul	Dormant	0.36	11.42
		Santiaguito	Dormant	1.09	34.56
		Semeru	Dormant	0.72	22.83
		Shiveluch	Dormant	2.08	65.96
		Soufriere Hills	Dormant	1.8	57.08
		Tinakula	Dormant	0.16	5.07
		Ulawun	Dormant	0.09	2.85
		Villarrica	Dormant	0.06	1.90
		Yasur	Dormant	0.2	6.34

Pasternak and Varekamp (1997)[14]	Crater lake heat budgets	Kerintji	Dormant		79
		Dempo	Dormant		142
		Kelut	Dormant		107
		Kawah Ijen	Dormant		244
		Keli Mutu TiN	Dormant		54
		Rincon Viejo	Dormant		105
		Golovnin	Dormant		100
		Ebeko	Dormant		69
		Zao	Dormant		177
		Narugo	Dormant		124
		Copahue	Dormant		173
		Cotacachi	Dormant		327
		Quilotoa	Dormant		72
		Ruapehu	Dormant		385
Chiodini et al. (2001)[18] ¥	Energy flux measured as diffusive soil heat transfer	Solfatara volcano	Dormant		138
Brown et al., (1991)[17]	Heat flux from the lake-filled crater.	Poas Crater Lake Volcano	Dormant		265
Brown et al., (1991) ✓	Volcano monitoring by microgravity and energy budget analysis	Poas Crater Lake Volcano	Dormant	200	500
Augustsdottir and Brantley (1994)[19]	One of the most active volcanoes in Iceland, covered by ice	Grimsvotn Volcano	Active		4250
Le Guern (1987)[20]	Pre-eruptive magma migration	Nyiragongo volcano	Active		16000
Baker and Massoth (1987) [21]	Hydrothermal vent in the Juan de Fuca ridge	S. Symmetrical Segment	Sub-sea		1700 ± 1000
Baker and Massoth (1987) [21]	Hydrothermal vent in the Juan de Fuca ridge	Endeavor Segment	Sub-sea		580 ± 351
Jean-Baptiste et al (1998) [22]	East Pacific Rise	Lucky Strike Vent	Sub-sea		3800 ± 1200

¥ Considered dormant and quiescent
✓ Includes crater lake but not Geyser or fumarole production, which may be 15 to 1000 MW, intermittently.

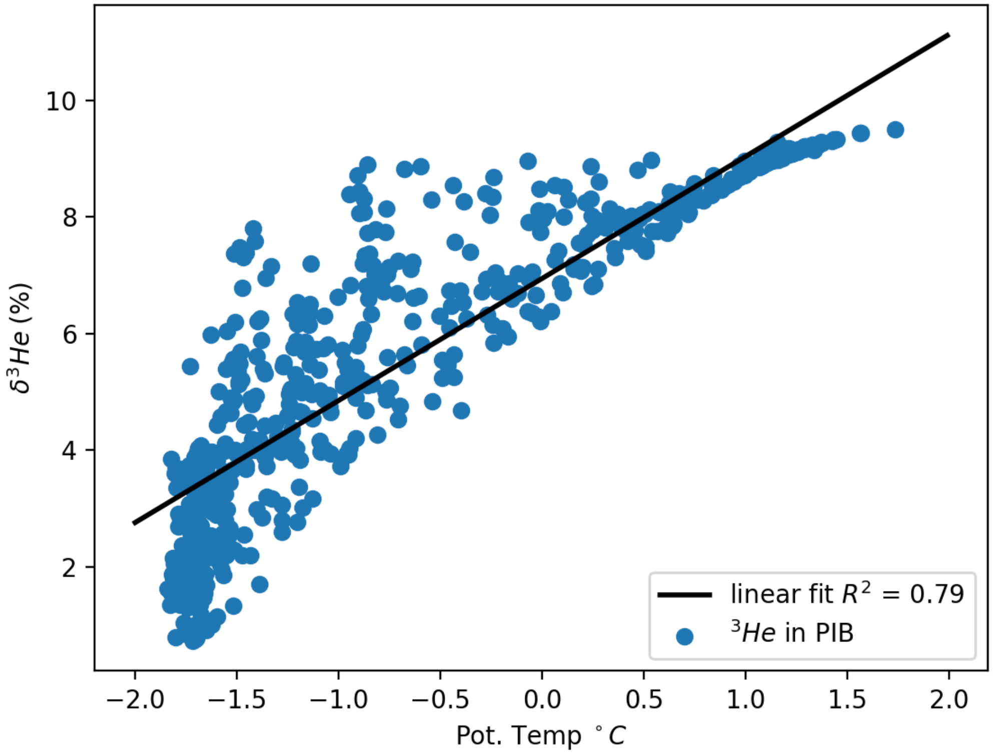

Supplementary Figure 1. The linear fit to OMP model of δ³He measurements in Pine Island Bay and to the observed potential temperature. The slope and intercept are m = 2.13, and b = 6.92, respectively.

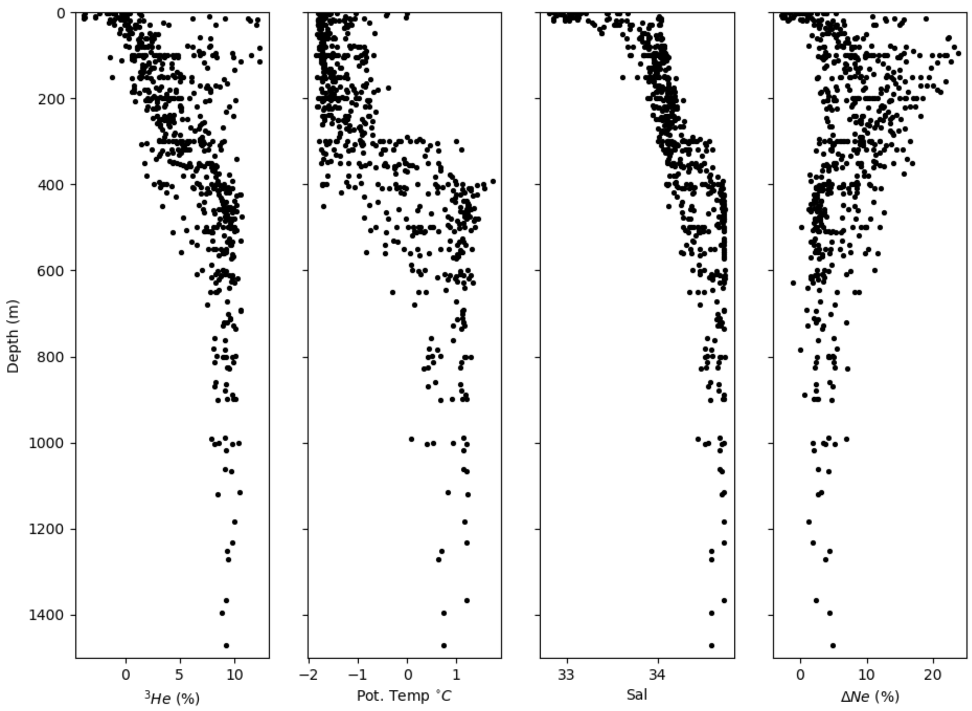

Supplementary Figure 2. Depth profiles of the 616 discrete samples that were collected during JR294 and NBP07-02 in the Amundsen Sea sector of the Southern Ocean. These samples were used as water mass tracers in the OMP linear water mass mixing calculation described in the M1 Methods section. Here, we express Neon as saturation anomaly ($\Delta Ne(\%) = \left(Ne_{obs} / Ne_{sat}(S,\theta) - 1 \right) \cdot 100$) to depict the excess neon dissolved in seawater, some of which can be attributed to glacial melt.

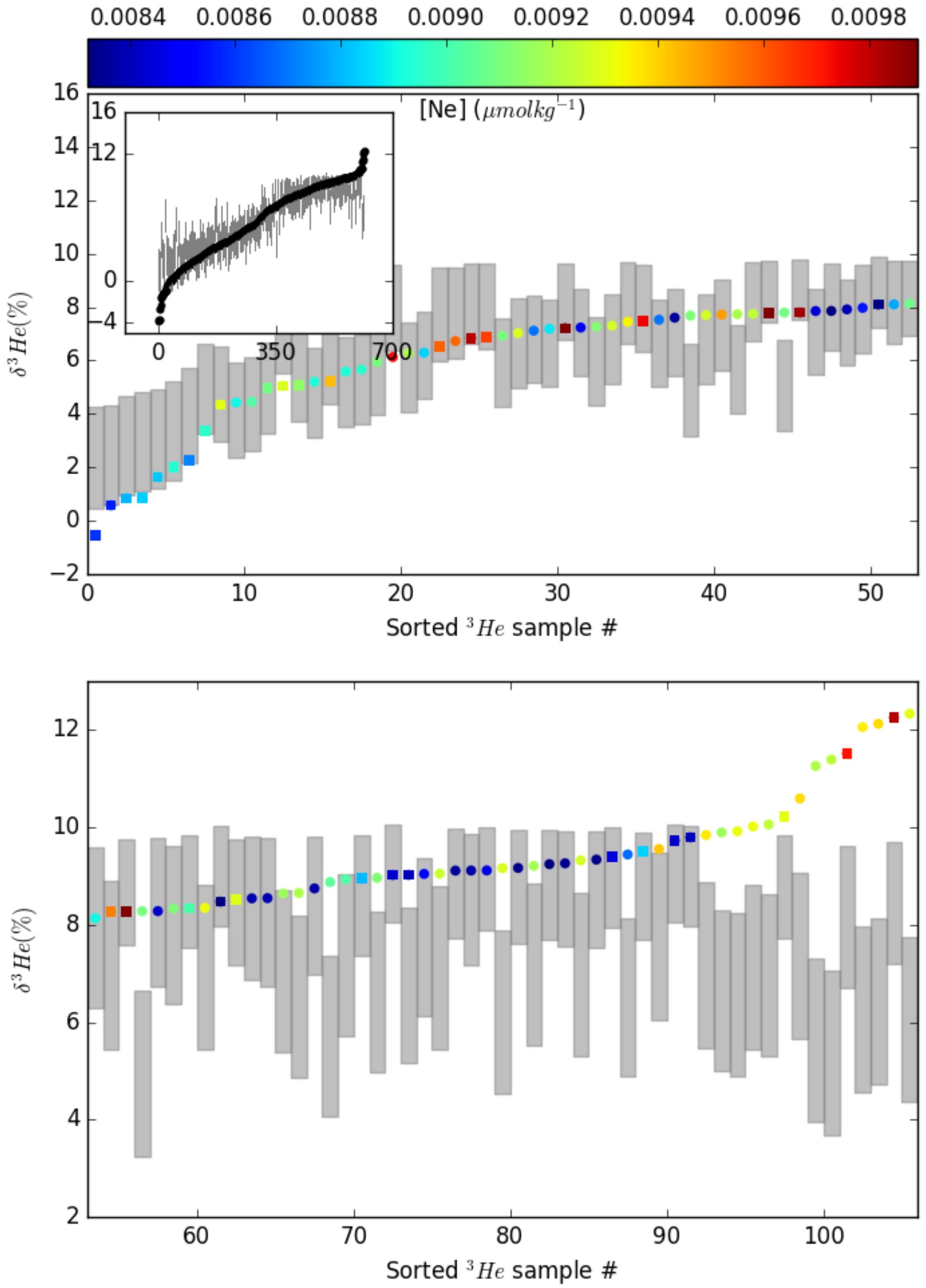

Supplementary Figure 3. Upper panel inset the black dots shows the N = 1610 δ^3He samples from the Amundsen Sea in 2007 (NBP07-02) and 2014 (JR294), sorted by increasing δ^3He values. The gray shaded bars indicate the 99% confidence interval

established using the Bootstrap resampling of the OMP mixing model. Upper and lower panels also show δ^3He samples sorted by increasing δ^3He, which depict each of the 106 samples from Pine Island Bay. The coloring of the symbols depicts neon concentration, which is a proxy for glacial meltwater. In total, 28 of the 106 δ^3He samples fall above the confidence region.

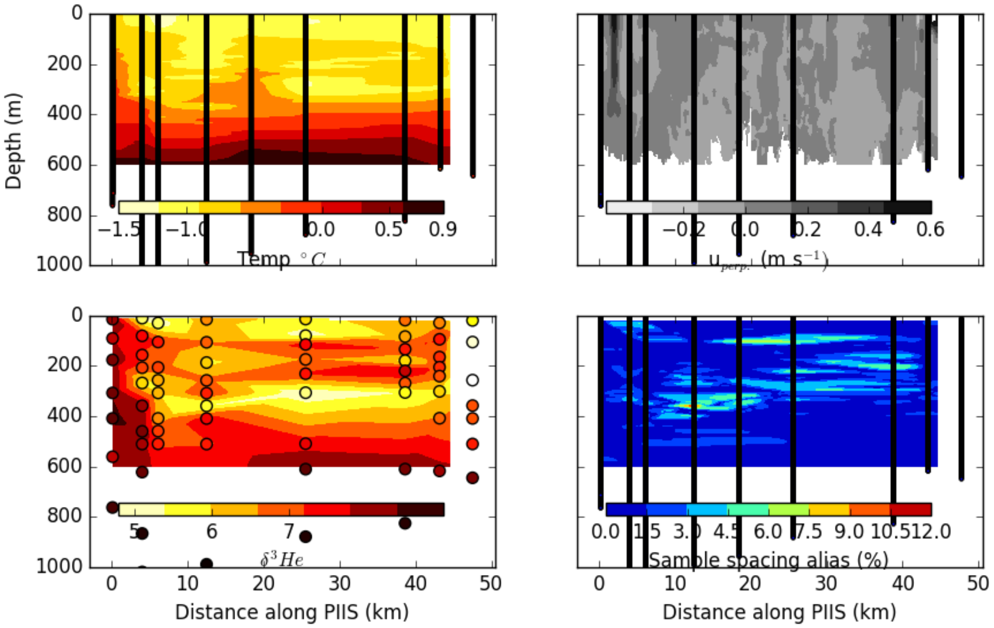

Supplementary Figure 4. Potential temperature from CTD (top left) and δ^3He (bottom left), which have been interpolated onto the gridded velocity field (u_{perp}) normal to the ice shelf face (top right). The difference in sample spacing produces local differences in the interpolation result. The bottom right panel shows difference in the directional derivative of interpolated δ^3He and interpolated temperature, expressed as % of full-scale δ^3He values.

Supplementary References:

1. Jenkins, W. J., Edmond, J. M. & Corliss, J. B. Excess 3He and 4He in Galapagos hydrothermal waters. *Nature* **272,** 156–158 (1978).

2. Schlosser, P., Roether, W. & Rohardt, G. Helium-3 balance of the upper layers of the northwestern Weddell sea. *Deep-Sea Res.* **34,** 365–377 (1987).

3. Winckler, G., Newton, R., Schlosser, P. & Crone, T. J. Mantle helium reveals Southern Ocean hydrothermal venting. *Geophys. Res. Lett.* **37,** L05601 (2010).

4. Hahm, D. *et al.* First hydrothermal discoveries on the Australian-Antarctic Ridge: Discharge sites, plume chemistry, and vent organisms. *Geochem. Geophys. Geosystems* **16,** 3061–3075 (2015).

5. Clarke, W. B., Beg, M. A. & Craig, H. Excess 3He in the sea: Evidence for terrestrial primodal helium. *Earth Planet. Sci. Lett.* **6,** 213–220 (1969).

6. Class, C. & Goldstein, S. L. Evolution of helium isotopes in the Earth's mantle. *Nature* **436,** 1107–1112 (2005).

7. O'Nions, R. K. & Oxburgh, E. R. Heat and helium in the Earth. *Nature* **306,** 429–431 (1983).

8. Hohmann, R., Schlosser, P. & Huber, B. Helium 3 and dissolved oxygen balances in the upper waters of the Weddell Sea: Implications for oceanic heat fluxes. *J. Geophys. Res.* **108,** (2003).

9. Well, R., Roether, W. & Stevens, D. P. An additional deep-water mass in Drake Passage as revealed by ^{3}He data. *Deep Sea Res.* **50,** 1079–1098 (2003).

10. Garabato, A. C. N., Stevens, D. P., Watson, A. J. & Roether, W. Short-circuiting of the overturning circulation in the Antarctic Circumpolar Current. *Nature* **447,** 194–197 (2007).

11. Orsi, A. H. & Wiederwohl, C. L. A recount of Ross Sea waters. *Deep-Sea Res. II* (2009). doi:10.1016/ j.dsr2.2008.10.033

12. Heywood, K. J. *et al.* Between the Devil and the Deep Blue Sea: The Role of the Amundsen Sea Continental Shelf in Exchanges Between Ocean and Ice Shelves. *Oceanography* **29,** (2016).
13. Dutrieux, P. *et al.* Strong sensitivity of Pine Island Ice-Shelf melting to climatic variability. *Science* **343,** (2014).
14. Pasternack, G. B. & Varekamp, J. C. Volcanic lake systematics I. Physical constraints. *Bull. Volcanol.* **58,** 528–538 (1997).
15. Wright, R. & Flynn, L. P. Space-based estimate of the volcanic heat flux into the atmosphere during 2001 and 2002. *Geology* **32,** 189–192 (2004).
16. Brown, G. *et al.* Energy budget analysis for Poas crater lake: implications for predicting volcanic activity. *Nature* **339,** 370–373 (1989).
17. BROWN, G. C., RYMER, H. & STEVENSON, D. Volcano monitoring by microgravity and energy budget analysis. *J. Geol. Soc.* **148,** 585 (1991).
18. Chiodini, G. *et al.* CO2 degassing and energy release at Solfatara volcano, Campi Flegrei, Italy. *J. Geophys. Res. Solid Earth* **106,** 16213–16221 (2001).
19. Ágústsdóttir, A. M. & Brantley, S. L. Volatile fluxes integrated over four decades at Grímsvötn volcano, Iceland. *J. Geophys. Res. Solid Earth* **99,** 9505–9522 (1994).
20. Le Guern, F. Mechanism of energy transfer in the lava lake of Niragongo (Zaire), 1959–1977. *J. Volcanol. Geotherm. Res.* **31,** 17–31 (1987).
21. Baker, E. T. & Massoth, G. J. Characteristics of hydrothermal plumes from two vent fields on the Juan de Fuca Ridge, northeast Pacific Ocean. *Earth Planet. Sci. Lett.* **85,** 59–73 (1987).

22. Jean-Baptiste, P. *et al.* Mantle 3He in hydrothermal vents and plume of the Lucky Strike site (MAR 37°17′N) and associated geothermal heat flux. *Earth Planet. Sci. Lett.* **157,** 69–77 (1998).

23. Jean-Baptiste, P., Fourre, E., German, J.-L. & Radford-Knoery, J. Helium isotopes at the Rainbow hydrothermal site (Mid-Atlantic Ridge, 361140N). *Earth Planet. Sci. Lett.* **221,** 325–335 (2004).

24. Lupton, J. E., Baker, E. T. & Massoth, G. J. Variable 3He/heat ratios in submarine hydrothermal systems: evidence from two plumes over the Juan de Fuca ridge. *Nature* **337,** 161–164 (1989).

25. Lupton, J. E., Baker, E. T. & Massoth, G. J. Helium, heat, and the generation of hydrothermal event plumes at mid-ocean ridges. *Earth Planet. Sci. Lett.* **171,** 343–350 (1999).

26. Jenkins, W. J., Lott III, D. E., Longworth, B. E., Curtice, J. M. & Cahill, K. L. The distributions of helium isotopes and tritium along the U.S. GEOTRACES North Atlantic sections (GEOTRACES GAO3). *GEOTRACES GA-03 - US GEOTRACES N. Atl. Transect* **116,** 21–28 (2015).
